# Sigma-1 receptor directly interacts with Rac1-GTPase in the brain mitochondria

**DOI:** 10.1186/s12858-015-0040-y

**Published:** 2015-04-30

**Authors:** Nino Natsvlishvili, Nino Goguadze, Elene Zhuravliova, David Mikeladze

**Affiliations:** Institute of Chemical Biology, Ilia State University, 3/5 Cholokashvili av, Tbilisi, 0162 Georgia; Department of Biochemistry, I.Beritashvili Center of Experimental Biomedicine, 14 Gotua st, Tbilisi, 0160 Georgia

**Keywords:** Mitochondria, Mitochondria-associated membranes, Sigma-receptor, Rac1, ROS

## Abstract

**Background:**

Small Rho-GTPases are critical mediators of neuronal plasticity and are involved in the pathogenesis of several psychiatric and neurological disorders. Rac-GTPase forms a multiprotein complex with upstream and downstream regulators that are essential for the spatiotemporal transmission of Rac signaling. The sigma-1 receptor (Sig1R) is a ligand-regulated membrane protein chaperone, and multiprotein complex assembly is essential to sigma-receptor function.

**Results:**

Using immunoprecipitation techniques, we have shown that in mitochondrial membranes Sig1R could directly interact with Rac1. Besides Rac1, the Sig1R forms complexes with inositol 1,4,5-trisphosphate receptor and Bcl2, suggesting that mitochondrial associated membranes (MAM) are involved in this macromolecular complex formation. Assembly of this complex is ligand-specific and depends on the presence of sigma agonist/antagonist, as well as on the presence of GTP/GDP. Treatment of mitochondrial membranes with (+)-pentazocine leads to the (+)-pentazocine-sensitive phosphorylation of Bad and the pentazocine-sensitive NADPH-dependent production of ROS.

**Conclusion:**

We suggest that Sig1R through Rac1 signaling induces mild oxidative stress that possibly is involved in the regulation of neuroplasticity, as well as in the prevention of apoptosis and autophagy.

## Background

The sigma-1 receptor (Sig1R) is a member of a family of membrane-associated proteins that are found in the mammalian nervous system and peripheral tissues, including the immune and endocrine systems [1]. It has been suggested that Sig1R may be involved in diseases of the central nervous system (CNS) including amnesia, schizophrenia, depression, Alzheimer’s disease, and addiction [2,3]. Several CNS drugs show high to moderate affinities for Sig1R, including antipsychotics, opioids, antidepressants, antagonists of muscarinic, D2-dopamine and NMDA receptors, monoamine transporters inhibitors, selective serotonin reuptake inhibitors and monoamine oxidase inhibitors [4,5]. The Sig1R ligands show anti-amnesic and neuroprotective effects in a large variety of animal models; block neurodegeneration and regulate neuritogenesis [6].

Sig1R is localized in mitochondria-associated endoplasmic reticulum (ER) membranes (MAM), which are sites for the regulation of mitochondrial bioenergetics via ER calcium release [1]. This receptor could modulate a variety of intracellular signal transduction pathways through protein-protein interactions and could be associated with various proteins, including inositol 1,4,5-trisphosphate (IP3) receptor (IP3R), IRE, BiP, ankyrin, etc. in the MAM. In resting condition, Sig1R resides with the ER-resident chaperone BiP. It has been shown that under ER stress or via agonist stimulation Sig1R dissociates from BiP and binds IP3R, enhancing calcium entry into mitochondria [1,7]. This calcium spike evokes redox reactions and ATP productions by regulating Ca^2+^-dependent enzymes in the TCA cycle, whereas mitochondrial calcium overloading leads to apoptosis [8,9]. Furthermore, during the activation Sig1R translocates to the other cell compartments and binds to different membrane proteins, including ion channels, kinases, G-protein coupled receptors (GPCRs), etc. [3,10].

Small Rho GTPases (e.g. Rho, Rac, CDC42) have been implicated in the neuropathogenesis of several psychiatric and neurological disorders and are critical mediators of neuronal growth cone dynamics, dendritic spine formation and axonal path finding [11]. Typically, Rac and its downstream effectors promote neuronal survival while Rho and its downstream effectors are capable of inducing neuronal apoptosis. Rho family GTPases contain a ∼ 200 amino acid residue Dbl homology (DH) domain and an adjacent, C-terminal, 120 residue pleckstrin homology (PH) domain. Thus, these proteins can form multiprotein complexes that are essential for the transmission of downstream signaling pathways. The main effector of Rac in the neurons is p21-activated kinase (PAK). Rac-dependent activation of PAK promotes survival of neurons through stimulation of the mitogen-activated protein kinase (MAPK) and phosphatidyl inositol-3 kinase (PI3K)/Akt pathways that inhibits the activity of pro-apoptotic members (e.g., Bad) and enhances the expression of pro-survival members (e.g., Bcl-xL) of the Bcl-2 family of proteins [12-14].

A growing body of evidence suggests that reactive oxygen species (ROS) presented at low or moderate levels act as secondary messengers to regulate cell growth, survival, and proliferation [15,16]. Mitochondria are the major intracellular source of ROS. ROS produced by mitochondria have been shown to play a significant role in intracellular signaling. Among other proteins, Bcl-2 is capable of regulating mitochondrial ROS. In addition to its canonical anti-apoptotic activity, Bcl-2 has been implicated in mitochondrial ROS regulation by its effect on interaction with the Rac1 [17,18]. Silencing and functional inhibition of Rac1 protein block the Bcl-2–mediated enhancement of intracellular superoxide levels. Rac1 is known to be involved in the assembly and activation of NADPH oxidase complex, leading to ROS production [19]. It should be noted that Sig1Rs regulate the neuroplasticity via a potential ER-mitochondrion-Rac1 pathway, apparently through the production of the ROS that doesn’t involve Ca^2+^ signaling [20]. However, it is still not clear how Sig1Rs modulate free radicals or reactive oxygen species in the neuron. We hypothesize that Sig1R could regulate the production of ROS through ER-mitochondrial Rac1 system. This system could promote a mild pro-oxidant milieu for cellular signaling and induce plastic transformation in neurons. We report here that Rac1 interacts with the Sig1R in a protein complex, containing IP3R, BiP and Bcl2. This complex in the presence of (+)-pentazocine could induce ROS production through activation of NADPH-oxidase.

## Methods

### Materials

Reagents and antibodies were obtained from the following sources: anti-OPRS1 (Sig1R) antibody (Abcam, UK), anti-IP3 receptor antibody (Abcam, UK), Anti-GRP78 (BiP) antibody (Abcam, UK), anti-mitofusin 2 antibody (Abcam, UK), anti-Rac1 antibody (Santa Cruz, USA), anti-Bcl 2 antibody (Santa Cruz, USA), GppNHp (Sigma-Aldrich, USA), GDP (Sigma-Aldrich, USA), pentazocine (Sigma-Aldrich, USA), haloperidol (Sigma-Aldrich, USA).

### Isolation of brain mitochondria

Isolation of cortical non-synaptosomal bovine brain mitochondria was achieved by using a discontinuous Percoll gradient [21]. Briefly, brain cortex was removed and gently homogenized in 10 fold volume of isolation buffer (5 mM HEPES, 225 mM mannitol, 75 mM sucrose, 1 mM EGTA, pH 7.5). Homogenate was centrifuged at 1,250 × g for 5 minutes. Obtained supernatant was immediately centrifuged at 21,000 × g for 10 min. The pellet was resuspended in cold 15% Percoll solution and layered on the gradient of 23 % and 40 % Percoll solutions. The samples were centrifuged at 25,000 × g for 8 minutes, and lower interphase was collected, washed twice with isolation buffer and resuspended in isolation buffer without EGTA. All steps were carried out under ice-cold conditions.

Mitochondria were tested for oxygen consumption in the state 3 of respiration in the presence of NAD ^+^-linked substrates (glutamate + malate). Respiration rate was 400–500 nmol O_2_/min per mg of protein.

### NADPH oxidase assay

NADPH oxidase activity was determined in the incubation medium contained bovine cortex mitochondria (60–120 μg of protein), 10 mM phosphate buffer, 130 mM NaCl, 1 mM EGTA, 10 μM flavin adenine dinucleotide (FAD), 2 mM NaN_3_, 1 μM (+)-pentazocine or/and 1 μM haloperidol, 50 μM oxidized cytochrome *c*, 200 units of superoxide dismutase (SOD) in parallel samples. Reactions were initiated by the addition of NADPH to a final concentration of 200 μM. After 30 min, activity was measured as the SOD-inhibitable increase in absorbance at 550 nm (Ε_mM_ = 21) [22].

### Immunoprecipitation and Western blotting

The freshly isolated mitochondrial pellet (0.25 mg for each sample) was resuspended in isolation buffer (5 mM HEPES, 225 mM mannitol, 75 mM sucrose) and mitochondria was incubated either with 1 μM (+)-pentazocine or 1 μM haloperidol, or with 10 μM Guanosine 5′-[β,γ-imido]triphosphate (Gpp(NH)p or with 10 μM GDP at 4°C for 60 min. After incubation mitochondria was precipitated by centrifugation at 20,000 × g for 20 min, pellets were resuspended in ice-cold lysis buffer (20 mM Tris–HCl pH 8.0, 137 mM NaCl, 10% glycerol, 1% Triton X-100, 2 mM EDTA), incubated for 60 min at 4°C. The unsolubilized material was removed by centrifugation (60 min at 20,000 × *g*). The supernatants were incubated with antibody-bound protein A/G-Agarose beads overnight at 4°C. After washing, the protein A/G-Agarose pellets were resuspended in 100 mM glycine, pH 3.0, for 10 s, and then a pretitrated volume of 1.0 M Tris, pH 9.5, was added to adjust the pH to 7.4. Protein complexes in the supernatants (2,500 × g, 10 min) were then analyzed by Western blotting.

For immunoblotting experiments, 50 μg of protein was separated by SDS-polyacrylamide gel electrophoresis and transferred to nitrocellulose sheets. After blocking with blocking buffer (5% bovine serum albumin, 0.05% Tween 20 in Tris–HCl-buffered saline), the sheets were incubated with primary antibodies in the blocking solution. Labeled bands were visualized using enhanced chemiluminescence (Amersham, California, USA) and analyzed by densitometric scanning. The content of proteins was quantified by the intensity of the bands, which is linear to the quantity of samples applied to the gel.

### Bad phosphorylation assay

Bad phosphorylation was determined by PhosphoTracer BAD(pSer112) + Total BAD ELISA kit (Abcam, UK). Briefly, the freshly isolated mitochondrial pellet (0.25 mg for each sample) was resuspended in isolation buffer (5 mM HEPES, 225 mM mannitol, 75 mM sucrose) and mitochondria were incubated either with 1 μM (+)-pentazocine, with 1 μM haloperidol or both ligands at 4°C for 60 min. After incubation mitochondria were precipitated by centrifugation at 20,000 × g for 20 min, pellets were resuspended in ice-cold lysis buffer (20 mM Tris–HCl pH 8.0, 137 mM NaCl, 10% glycerol, 1% Triton X-100, 2 mM EDTA) and unsolubilized material was removed by centrifugation (60 min at 20 000 × *g*). Mitochondrial extracts were subjected to a PhosphoTracer assay plate, and total BAD and BAD(pSer112) were analyzed according to manufacturer’s protocols.

Protein concentration was determined using a dye-binding method (Bio-Rad).

### Statistical analysis

All experiments were performed in triplicates and repeated at least twice. Results are expressed as mean ± the standard error of the mean (SEM). For statistical analysis, a t-test was used. A difference between experimental groups was considered to be significant when P < 0.05.

## Results and discussion

### Rac1 interacts with Sig1R through the multiprotein complex

**Figure 1:**
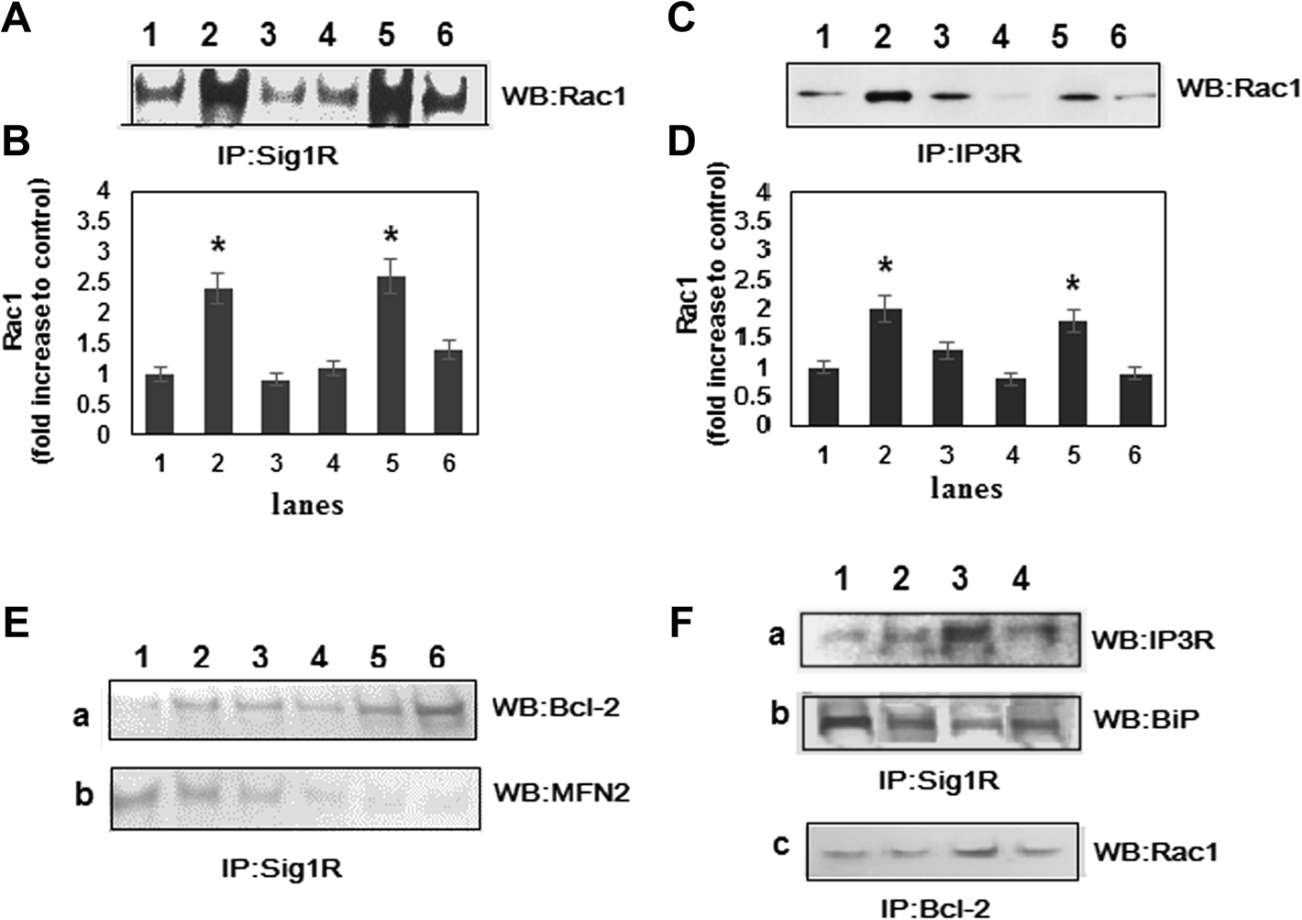
Effect of pentazocine (P), haloperidol (H) and guanine nucleotides on the interaction between the Sig1R, Rac1, and Bcl-2. Mitochondria were incubated with sigma ligands and guanine nucleotides as described in Material and methods. Mitochondrial extracts were immunoprecipitated with anti-Sig1R (IP: Sig1R, **A**, **B**, **E**, **Fa**, **Fb)**, with anti-IP3R (IP: IP3R, C,D) or anti-Bcl2 (IP: Bcl2, Fc) antibodies, the immunoprecipitate was subjected to SDS-PAGE and transferred. The blots were then probed to Western analysis with antibodies against Rac1(Wb: Rac1, A,C,Fc), Bcl2(Wb:Bcl2, Ea), MFN2(Wb:MFN2, Eb), IP3R (Wb:IP3R, Fa) and BiP (Wb:BiP, Fb). Results are representative of three independent experiments. **(A, C, E, )** Lane 1 – control, no additions; lane 2 – plus GppNp; lane 3 – plus GDP; lane 4 – plus haloperidol; lane 5- plus (+)-pentazocine; lane 6- plus haloperidol and (+)-pentazocine. **(F)** Lane 1 – no additions; lane 2 – plus haloperidol, lane 3 – plus (+)-pentazocine; lane 4 - plus haloperidol and (+)-pentazocine. **(B)** Quantification of Rac1 blots shown in A; n = 3. *P < 0.05, compared with control levels. **(D)** Quantification of Rac1 blots shown in C; n = 3. *P < 0.05, compared with control levels.

To investigate the association of Sig1R with Rac1 and other mitochondrial proteins immunoprecipitation experiments were performed. Mitochondria were incubated with 10 μM haloperidol (sigma-1 receptor antagonist), with 10 μM (+)-pentazocine (sigma-1 receptor selective agonist) or both ligands for 60 min at 4°C. These concentrations of drugs were in the clinically relevant range, and the affinity of both drugs to Sig1R was similar (6.5nM for haloperidol and 1.7nM, for (+)-pentazocine) [23]. Like other small G proteins, Rac1 cycles between an inactive, GDP-bound state and an active, GTP-bound state. To determine the role of active and inactive state in the formation of the macromolecular complex, mitochondria were incubated with non-hydrolysable analogs of GTP - GppNHp or GDP. Mitochondrial extracts were immunoprecipitated with agarose-conjugated anti-Sig1R, anti-IP3R or anti-Bcl-2 antibodies and eluted proteins were subjected to Western blot analysis (Figure 1).

Western blot of mitochondrial proteins has shown that Sig1R is immunoprecipitated with the Rac1. The association between these proteins is increased in the presence of (+)-pentazocine (Figure 1A and 1B, lane 5), and the formation of the immunoprecipitate is decreased in case of simultaneous treatment of mitochondrial proteins by (+)-pentazocine and haloperidol (Figure 1A and 1B, lane 6). Interaction between Sig1R and Rac1 is also enhanced in the presence of GppNHp (Figure 1A and 1B, lane 2), suggesting that active Rac1 directly or indirectly interacts with Sig1R.

Taking into account that Sig1R forms a complex with the various proteins in the MAM, we decided to analyze the binding of Sig1R with the IP3R, BiP and Bcl-2. The (+)-pentazocine-dependent association of Sig1R with IP3R was found when immunoprecipitation was performed with Sig1R antibodies and immunoreactivity was detected by anti-IP3R. (Figure 1Fa, lane 3). Moreover, vice versa, (+)-pentazocine-dependent dissociation of Sig1R from BiP was demonstrated when mitochondrial extracts were immunoprecipitated by anti-Sig1R and immunoreactivity was tested by anti-BiP (Figure 1Fb, lane 3). Haloperidol, in both cases, eliminated the effects of (+)-pentazocine (Figure 1Fa and 1Fb, lane 4), confirming the observation that sigma-ligands cause the redistribution of Sig1R between IP3R and BiP [1].

It is interesting that such ligand-specific interactions between Sig1R and Bcl-2 were not found when immunoprecipitations were performed by anti-Sig1R (Figure 1Ea lanes 4–6). However, when mitochondrial extracts were immunoprecipitated by anti-Bcl2, and protein bands were detected by anti-Rac1, (+)-pentazocine-dependent increase of immunoreactivity was found (Figure 1Fc lane 3). These data demonstrate that Sig1R is not directly involved in the formation of the Bcl-2-Rac1 complex. Furthermore, we have found that Rac1 could be associated with IP3R, and this association depends on the presence of SigR1-ligands and guanine nucleotides (Figure 1C and 1D, lanes 1–6). Altogether this suggests that Rac1 specifically binds to the IP3R and this interaction is regulated by both, sigma ligands, and guanine nucleotides.

It should be noted that mitofusin-2 (MFN2), one of the major GTP-binding protein in MAM, was also immunoprecipitated by anti-Sig1R, however, in this case, neither sigma ligands nor guanine nucleotides changed proteins’ association (Figure 1Eb). In addition, we have found that neither K-Ras nor H-Ras bind to the Sig1R (data not shown).

### Sigma ligands change the activities of Rac1 down-stream effectors

**Figure 2:**
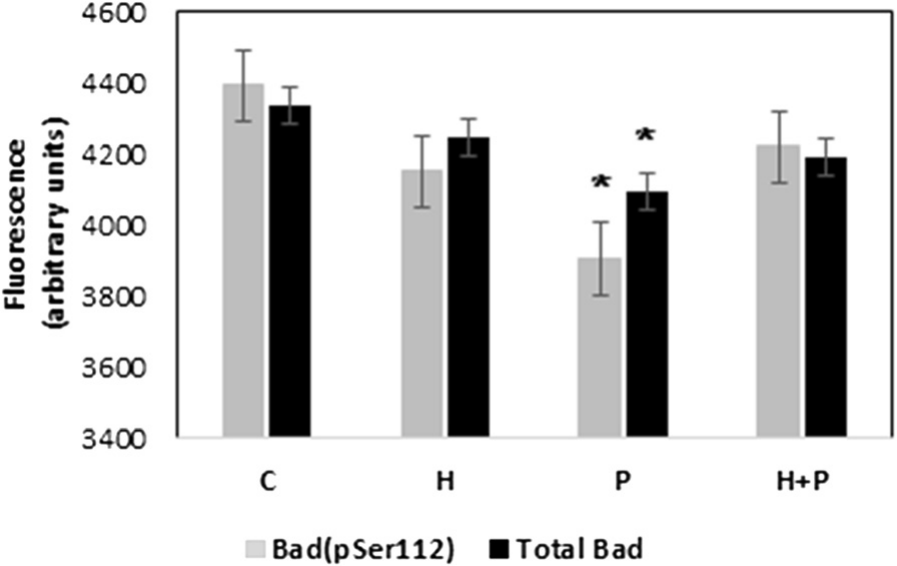
Effects of (+)-pentazocine and haloperidol on the content of total Bad and Bad(pSer112) in mitochondrial lysates. Mitochondria were incubated with haloperidol (H), with (+)-pentazocine (P) or with both ligands (H + P) as described in Material and methods. Mitochondrial lysates were subjected to the analyzes of total Bad and Bad(pSer112). Values in the ELISAs (means ± SD) are representative of three experiments and are reported as arbitrary units. Each assay was performed at least in duplicate. *P < 0.05 compared with the control (C).

p21-activated kinases are the best-characterized downstream effectors of the Rac1. PAK directly or indirectly, through other protein kinases (i.e. Raf, Akt) can phosphorylate various regulatory proteins, including pro-apoptotic Bad [24]. Phosphorylation of Bad at S112 and S136 decreases Bad/Bcl-2 complex formation, which results in increased cell survival [25]. Thus, one of the targets of Sig1R-Rac complex in the mitochondria could be the PAK/Bad pathway. To determine whether sigma ligands change Bad phosphorylation, mitochondria were incubated with sigma ligands, and Bad-pSer112 and total Bad were identified in the mitochondrial extracts. Our results have shown that (+)-pentazocine decreased the immunoreactivity of mitochondria against both, Bad(pSer112) and total Bad, suggesting that the sigma agonist causes dissociation of Bad from mitochondria. In this case, haloperidol eliminated the effect of pentazocine (Figure 2).

**Figure 3:**
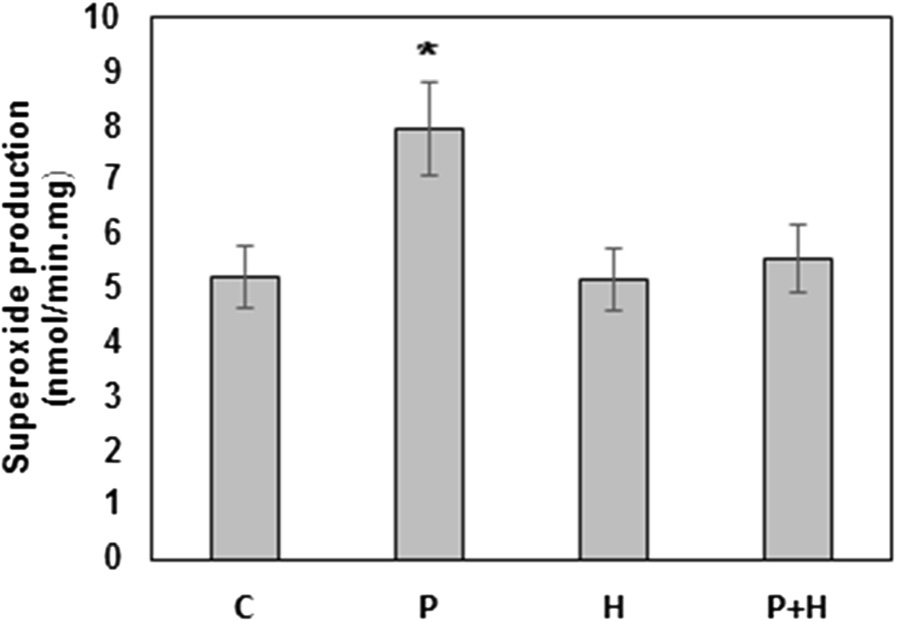
Effects of pentazocine and haloperidol on the NADPH oxidase activity. Mitochondria were incubated with haloperidol (H), with (+)-pentazocine (P) or with both ligands (H + P) and NADPH oxidase activity determined as described in Material and methods. Data represented are mean ± SEM of results from four separate experiments performed in duplicate. *P < 0.05, t test compared with corresponding control (C).

Rac1 is known to be involved in the assembly and activation of NADPH oxidase. NADPH oxidases (NOX) are a family of enzyme complexes whose primary function is to catalyze the transfer of electrons from NADPH to molecular oxygen, generating superoxide anion and H_2_O_2_. It has been reported that isoforms of NADPH oxidase are expressed in mitochondria. However, the molecular mechanisms underlying the interplay between the NADPH oxidases and the mitochondria remain undefined [26]. To determine whether sigma ligands change NADPH oxidase activity, we analyzed the production of ROS in mitochondrial suspension. We have found that the production of ROS was increased in the presence of pentazocine, and haloperidol eliminated the effect of (+)-pentazocine (Figure 3).

Rho family GTPases are key players in neuronal development, neuronal survival and neurodegeneration [14]. Rac activation typically promotes neuronal survival while Rho activation often elicits neuronal death [13,27]. In most cases, Rac-GTPase forms a multiprotein complex with upstream and downstream regulators that are essential for the spatiotemporal transmission of Rac signaling [11]. Rac1 primarily activates PAK-s increasing phosphorylation of various regulatory and cytoskeletal proteins [12,13]. Besides, Rac forms complexes with p67phox, regulates redox signaling through activation of NADPH oxidase [19] and could initiate ER-stress [28]. Rac1 may also be found at the mitochondrial membrane where it interacts with Bcl-2 through the BH3 domain and initiates mild oxidative stress [17].

Sig1R is neuroprotective in stroke and cerebral ischemia, and has been linked to schizophrenia, Alzheimer’s disease, depression and drug addiction [2,3]. The activity of Sig1R is regulated by various exogenous ligands, including antipsychotic (e.g. haloperidol) and psychotomimetic (e.g. pentazocine) drugs [29]. The protein-protein interaction is essential in sigma-receptor function, and like Rac1, Sig1Rs are involved in the morphogenesis of neurons [5]. This data suggests that the pathways of regulation of these proteins may cross at various stages of survival and development of neurons.

Using immunoprecipitation techniques, we have shown for the first time that Sig1R could directly interact with Rac1. This interaction is a ligand-specific since the complex between Sig1R and Rac1 is formed only in the presence of (+)-pentazocine and the complex formation between these proteins is abrogated by antagonist - haloperidol. In addition, our results have shown that the active, GTP associated Rac1 is also capable to form a complex with Sig1R. In our opinion, this complex is assembled on the IP3R, which can interact with a broad range of proteins in MAM [30]. Our experiments have shown that (+)-pentazocine reduces the binding of Sig1R to the BiP and increases its association with IP3R, whereas haloperidol changes this redistribution. These data agree with the accepted consideration that after agonist’s stimulation, Sig1Rs dissociate from BiP and act on IP3Rs, leading to a prolonged Ca^2+^ signaling into mitochondria [1].

Our results have also shown that Rac1 forms multiprotein complex with Bcl-2 (Figure 1Fc). BH3 domain of Bcl-2 is necessary for interaction with Rac1, and this interaction is critical for the pro-oxidant activity of Bcl-2 [17]. It is important that anti-apoptotic Bcl-2 family members form a macromolecular complex with the IP3Rs in the ER [31]. The interaction between these proteins sensitizes IP3Rs to low IP3 concentrations leading to the decline in Ca^2+^-store and the induction of small oscillatory Ca^2+^ signals [32]. As a consequence, cells are protected against apoptosis. Besides Bcl-2 family members, various proteins, involved in the regulation of IP3-induced Ca^2+^release from ER, could directly bind to the IP3R.

To assess the activity of Rac, we have investigated the effect of sigma ligands on the possible downstream effector systems. The activity of two best-characterized target systems of Rac (PAK-protein kinase and NADPH-oxidase), were analyzed in the mitochondrial fraction. PAK could directly phosphorylate various regulatory proteins, including mitochondrial Bad [24]. Our results have shown that (+)-pentazocine induces the phosphorylation and subsequent dissociation of Bad from mitochondria. Besides, we have found that (+)-pentazocine increases the NADPH-dependent production of ROS, which is inhibited by haloperidol. Given that pro-apoptotic Bad is dissociated from Bcl-2 after phosphorylation, we conclude that activation of sigma receptor by an agonist leads to the release of BH3-only protein from mitochondria (or MAM). This relocation of Bad may be important for the prevention of apoptosis and autophagy [33]. The dissociation of Bad from Bcl2 could facilitate Rac1-Bcl2 interaction and induce mild non-oxidative ROS formation [18] through mitochondrial NADPH-oxidase. This mechanism may be involved in the regulation of neuroplasticity by sigma ligands [20].

Thus, we suggest that through IP3R/Sig1R/Bcl-2/Rac1 multiprotein complex, sigma agonists could induce mild oxidative alterations in mitochondria that may be crucial for cell homeostasis. However, further studies are clearly needed to characterize this interaction. Taking into account the involvement of Rac1 and Sig1R in cancer cell metabolism, as well as in neurological disorders, understanding the molecular basis of their interaction may have implications for a therapeutic approach, based on modulation of ER-stress.

## Abbreviations

ATP: Adenosine triphosphate
CDC42: Cell division control protein 42 homolog
CNS: Central neural system
EDTA: Ethylenediaminetetraacetic acid
EGTA: Ethylene glycol tetraacetic acid
ER: Endoplasmic reticulum
FAD: Flavin adenine dinucleotide
GDP: Guanosine diphosphate
GPCR: G protein–coupled receptor
Gpp(NH)p: Guanosine 5′-[β,γ-imido]triphosphate
GTP: Guanosine triphosphate
HEPES: 4-(2-hydroxyethyl)-1-piperazineethanesulfonic acid
IP3R: Inositol trisphosphate receptor
IRE: Iron-responsive element
MAM: Mitochondria-associated ER membrane
MAPK: Mitogen-activated protein kinase
MFN2: Mitofusin-2
NADPH: Nicotinamide adenine dinucleotide phosphate
NMDA: N-methyl-D-aspartate
NOX: NADPH oxidase
PAK: p21-activated kinase
PI3K: Phosphatidylinositol-4,5-bisphosphate 3-kinase
ROS: Reactive oxygen species
Sig1R: Sigma-1 receptor
SOD: Superoxide dismutase
TCA cycle: Tricarbon acid cycle
